# Profile of thyroidectomies in Brazil from 2010 to 2020 from a macro-regional perspective

**DOI:** 10.20945/2359-3997000000590

**Published:** 2023-01-25

**Authors:** Joyce Pantoja Braga, Lívia Guerreiro de Barros Bentes, Rafael Silva Lemos, Nyara Rodrigues Conde Almeida, Manuela Rodrigues Neiva Fernandes, Gabrielly Leite Andrade, Victor Matheus Mendonça de Araújo, Deivid Ramos dos Santos

**Affiliations:** 1 Universidade Federal do Pará Laboratório de Cirurgia Experimental Belém PA Brasil Universidade Federal do Pará (UFPA), Laboratório de Cirurgia Experimental, Belém, PA, Brasil.; 2 Universidade do Estado do Pará Laboratório de Cirurgia Experimental Belém PA Brasil Universidade do Estado do Pará (UEPA), Laboratório de Cirurgia Experimental, Belém, PA, Brasil.; 3 Centro Universitário do Estado do Pará Laboratório de Cirurgia Experimental Belém PA Brasil Centro Universitário do Estado do Pará (CESUPA), Laboratório de Cirurgia Experimental, Belém, PA, Brasil.

**Keywords:** Thyroidectomy, Epidemiology, thyroid nodules

## Abstract

**Objective:**

To describe the distribution profile of thyroidectomies in Brazil from 2010 to 2020 from a macro-regional perspective.

**Materials and methods:**

This is a retrospective, detailed and descriptive study built on secondary data obtained from the Hospital Information System of the Unified Health System (SIH/SUS). We organized the data in tables and grouped them according to the federative unit, macro-region, type of procedure, mortality rate, and year of performance. We performed statistical analysis using the χ2 test to assess the association between the variables, observing a P value of < 0.05 and a confidence interval of 95%.

**Results:**

From 2010 to 2020, 160 219 thyroidectomy surgeries were performed, of which 77 812 (48.56%) were total, 38 064 (23.76%) partial and 41 191 (25.70%) oncological.The Southeast was responsible for the largest share of procedures, with 70 745 (44.15%), followed by the Northeast with 43 887 (27.39%). In 2020, the procedure was less performed, with 9226 (5.75%) surgeries. The total mortality rate was 0.16% during the study period.

**Conclusion:**

We found that thyroidectomies are carried out mainly in the Southeastern, Northeastern, and Southern regions, and showed a downward trend in 2020, which may be related to the COVID-19 pandemic. In addition, total thyroidectomy is the most performed surgery, and the Northern region had the highest mortality rate.

## INTRODUCTION

The thyroid is an endocrine gland located in the anterior region of the neck with the function of producing and secreting hormones that regulate metabolism (1). It is susceptible to morphological abnormalities, such as the appearance of nodules and hormonal dysfunctions. These conditions directly interfere with the quality of life of individuals, as they generate clinical repercussions throughout the body, such as sleep disorders, fatigue, tachycardia, changes in temperature, and the gastrointestinal system, negatively affecting the quality of life (1,2).

Among the non-oncological conditions that affect the thyroid, nodules are frequent findings in clinical practice, with a prevalence of around 10% in the adult population, being more common in women than in men (2,3). Most of these nodules are benign, with the most common causes being hyperplastic nodules, cysts, thyroiditis, and adenomas; however, approximately 5% of cases represent malignancy, with a higher risk in men (3,4).

Another condition that affects this organ is thyroid cancer, the most frequent among endocrine neoplasms, with an incidence of 11-15 cases per 100,000 inhabitants in Brazil, the fifth most common malignant neoplasm in Brazilian women besides non-melanoma skin cancer (5,6). The most common causes are well-differentiated carcinomas (papillary and follicular), representing 95% of cases and having a better prognosis than poorly differentiated (medullary and anaplastic) (7,8).

Treatment for thyroid disorders can be clinical (drugs, chemotherapy, radiotherapy), surgical, or both (1,2). Thyroidectomy is the surgical procedure for the removal of the thyroid, which can be total when the gland is removed entirely, or partial when the resection is of a segment or lobe. It is also performed on the lymph nodes, which consist of cervical lymph node dissection (2–4). The main indications for surgery are cases of previous head and neck irradiation, nontoxic multinodular goiter, malignant tumor, diffuse toxic goiter, and autoimmune thyroiditis (3,9).

Due to the density of important vessels in the region, such as the carotid arteries and jugular veins, and contiguity with important structures for other organs, such as the pharynx, these procedures must be performed in specialized services. Trained professionals and their materials are needed, especially in cancer patients, to avoid complications such as hemorrhaging, aphonia, and respiratory failure; this makes it difficult to carry out these procedures outside large cities and capitals (9,10).

Therefore, despite the clinical and epidemiological relevance of the topic for a continental country, we found no recent research addressing the distribution profile of these surgeries in Brazil, which indicates a field that has been little explored in the literature. Thus, the objective of this work is to describe the distribution profile of thyroidectomies in Brazil from 2010 to 2020 from the perspective of a macro-regional cut.

## MATERIALS AND METHODS

We conducted this study with a retrospective, quantitative and descriptive approach based on secondary data obtained from the Hospital Information System of the Department of Informatics in the Unified Health System (SIH/SUS-Datasus). We analyzed all cases of patients in the public health system undergoing thyroidectomy, in the time series, which corresponds to the period from 2010 to 2020.

The research categorized the procedure according to the surgical technique and its respective code as partial thyroidectomy (0402010035), total thyroidectomy (0402010043), total thyroidectomy with lymph node dissection (0402010051), thyroidectomy with neck dissection in oncology (0416030122) and total thyroidectomy in oncology (0416030130 and 0416030270).

Thus, we organized the data in tables and grouped them according to the federative unit, macro-region, type of procedure, mortality rate, year of performance, and average value of procedures. The number of deaths consists of the number of hospitalizations discharged due to death, in the Hospital Admission Authorization (AIH) approved in the period. The mortality rate refers to the ratio between the number of deaths and the number of approved AIHs computed as hospitalizations in the period, multiplied by 100. The average value is the total approved value of production referring to the approved AIH in the period divided by the amount of approved AIH. To demonstrate the distribution of the incidence of procedures in the country per 10,000 inhabitants, we prepared a map of the federation units using the Tabwin 4.15 program available on Datasus, using the resident population of the states and federal district provided by Tabnet-Datasus.

We performed statistical analysis using the Bioestat 5.3 program with the non-parametric χ2 test to express the association between variables, observing a p-value < 0.05 and a confidence interval of 95%. As it is a public domain database, the project is exempt from submission to the Research Ethics Committee.

## RESULTS

**Figure 1:**
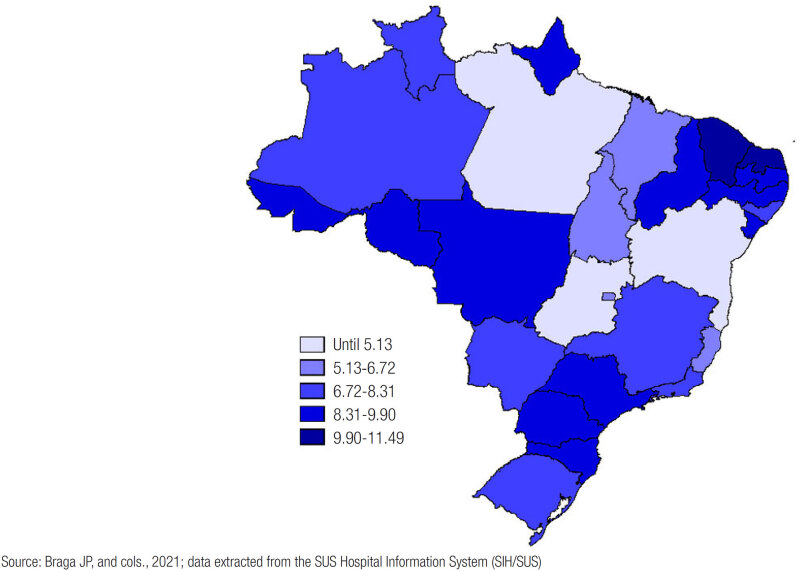
Coefficient of incidence of thyroidectomies per 10,000 inhabitants in Brazil from 2010 to 2020.

**Figure 2:**
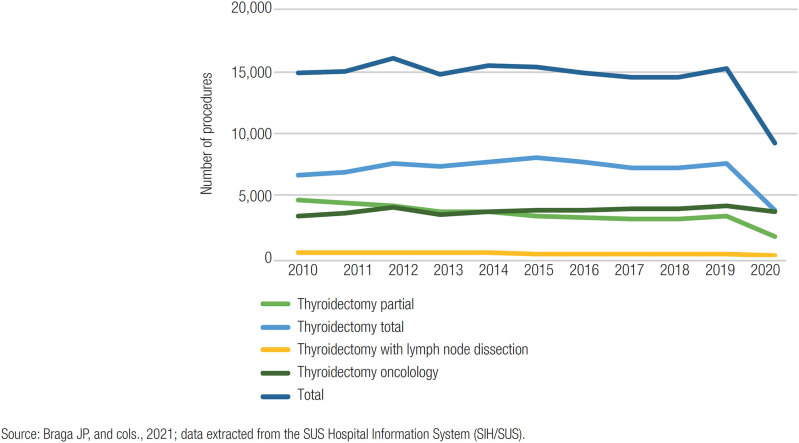
Number of thyroidectomies from 2010 to 2020, according to the year of the procedure.

From 2010 to 2020, 160,219 thyroidectomy surgeries were performed, of which 77,812 (48.56%) were total, 38,064 (23.76%) were partial and 41,191 (25.70%) were oncological. According to the regions, the South was responsible for the highest incidence of procedures, with a coefficient of 8.56 per 10,000 inhabitants, followed by the Southeast (8/10,000). The Northern region had the lowest rate (5.42/10,000), followed by the Center-West (Table 1 and Figure 1). Regarding the year in which the procedure was performed, 2012 had the highest number of thyroidectomies, with 16,110 (10.5%), while 2020 had fewer, with 9,226 (5.75%) surgeries (Figure 2 and Table 2).

**Table 1:** Distribution of cases and coefficient of the incidence of thyroidectomy surgeries by region between the years 2010 and 2020 reported in Brazil

Procedure (thyroidectomy)	CO	CI	N	CI	NO	CI	SE	CI	S	CI	Total	CI
Total	5,104	3.13	4,945	2.68	16,984	2.97	39,102	4.42	11,677	3.89	77,812	37.02
Partial	1,919	1.17	3,127	1.69	11,983	2.09	14,781	1.67	6,254	2.08	38,064	18.11
Total in oncology	2,558	1.56	1,480	0.80	12,146	2.12	14,251	1.61	6,294	2.09	36,729	17.47
Total with neck dissection	173	0.10	73	0.03	2,301	0.40	1,131	0.12	784	0.26	4,462	2.12
Total with lymph node dissection	153	0.09	367	0.19	473	0.08	1,480	0.16	679	0.22	3,152	1.49
Total	9,907	6.07	9,992	5.42	43,887	7.68	70,745	8.00	25,688	8.56	160,219	7.62

CO: Center-West; N: North; NO: Northeast; SE: Southeast; S: South; CI: coefficient of incidence x 10.000

Source: Braga JP, and cols., 2021; data extracted from the SUS Hospital Information System (SIH/SUS).

**Table 2:** Distribution of thyroidectomy cases per year from 2010 to 2020 reported in Brazil

Procedure (thyroidectomy)	2010	2011	2012	2013	2014	2015	2016	2017	2018	2019	2020	Total
Partial	4,673	4,359	4,111	3,667	3,643	3,271	3,150	3,083	3,100	3,346	1,661	38,064
Total	6,659	6,839	7,613	7,356	7,746	8,045	7,708	7,220	7,255	7,571	3,800	77,812
Total with lymph node dissection	356	322	366	349	343	248	240	295	246	270	117	3,152
Oncology	3,256	3,528	4,020	3,424	3,719	3,844	3,763	3,928	3,925	4,136	3,648	41,191
Total	14,944	15,048	16,110	14,796	15,451	15,408	14,861	14,526	14,526	15,323	9,226	160,219

Source: Braga JP, and cols., 2021; data extracted from the SUS Hospital Information System (SIH/SUS).

Regarding mortality (Table 3), 259 deaths from surgeries were reported during the study period, of which 112 (42.24%) were total thyroidectomies and 75 (28.95%) were oncological procedures. The total mortality rate was 0.16 per 100 procedures, with total thyroidectomy with lymph node dissection having the highest rate (0.41/100) and total thyroidectomy the lowest (0.14/100). Regarding the regions, the Southeast had the highest number of deaths, 124 (47.87%), and the North was responsible for the highest mortality rate, with 0.32 deaths per 100 thyroidectomies performed. In the temporal analysis, the year with the highest rate was 2010 (0.23/100) and the year with the lowest was 2019 (0.09), the other years had values between 0.13 and 0.19 for 100 surgeries.

**Table 3:** Distribution of the number of deaths and case mortality rate in thyroidectomy surgeries by region between the years 2010 and 2020 reported in Brazil

Procedure (thyroidectomy)	CO	TM	N	TM	NO	TM	SE	TM	S	TM	Total	TM
Partial	4	0.21	5	0.16	3	0.03	31	0.21	16	0.26	59	0.16
Total	5	0.10	12	0.24	21	0.12	57	0.15	17	0.15	112	0.14
Total with lymph node dissection	0	0	3	0.82	0	0	8	0.54	2	0.29	13	0.41
Oncology	3	0.10	12	0.77	16	0.11	28	0.18	16	0.22	75	0.18
Total	12	0.12	32	0.32	40	0.09	124	0.18	51	0.20	259	0.16

CO: Center-West; N: North; NO: Northeast; SE: Southeast; S: South; TM: mortality rate x 100.

Source: Braga JP, and cols., 2021; data extracted from the SUS Hospital Information System (SIH/SUS).

Regarding the average cost of the procedures, there is a direct proportion with the number of surgeries performed (Table 1 and Table 4). The global mean value of thyroidectomies was 1192.26 reais. The Northern macro-region had the lowest average cost of the analyzed procedures, with a value of 956.13 reais per surgery. The Northeast had the highest mean value for thyroidectomies, with 1316.49. As for the type of surgery, partial thyroidectomy had the lowest cost, at 507.67 reais, while oncological thyroidectomies had the highest overall average expenses (Table 4).

**Table 4:** Average value, in reais, by type of procedure and Brazilian regions between 2010 and 2020

Procedure (thyroidectomy)	CO	N	NO	SE	S	Total
Partial	515.61	514.35	478.56	520.62	527.1	507.67
Total	580.97	550.22	528.82	599.71	573.53	575.93
Total with lymph node dissection	929.9	951.29	903.09	1,001.35	880.67	951.31
Total with neck dissection in oncology	2,949.99	3,298.82	2,982.31	3,109.74	3,084.26	3,036.45
Total in oncology	3,071.96	3,131.41	2,945.11	2,995.52	3,079.68	3,004.07
Total	1,258.24	956.13	1.316.49	1.114.33	1,261.02	1,192.26

CO: Center-West; N: North; NO: Northeast; SE: Southeast; S: South; TM: mortality rate x 100.

Source: Braga JP, and cols., 2021; data extracted from the SUS Hospital Information System (SIH/SUS).

## DISCUSSION

Thyroidectomy is a surgery performed in all Brazilian macroregions and offered by the Unified Health System (SUS) in medium and high complexity centers, treating from non-oncological conditions, such as benign nodules with systemic and psychosocial repercussions on the patient's life, to oncological ones, leading to changes in the prognosis and survival of a complex condition (9).

The data collected in this study showed that non- cancer procedures were more frequently applied, considering that non-neoplastic conditions are the most common causes that lead to the need for surgical treatment (3–6). Such findings corroborate the research by Da Silva and cols. (2020) (11), which verified the incidence of benign findings in most patients undergoing the procedure. Non-oncological total thyroidectomy was the most used surgery, accounting for nearly half of the cases (48.56%) in the years 2010 to 2020, which corroborates the literature. This procedure is the most performed endocrine surgery worldwide, and is the most indicated in the guidelines for the treatment of thyroid disorders, as it causes fewer complications, reoperations, and recurrences (3,12–14).

In the regional analysis, there was a higher incidence of surgeries in the most developed regions, the Southeast and South because, historically, there is an unequal distribution of medical professionals and health conditions (15). According to the Datasus, the Southeast is the macro-region with the highest percentage of citizens using medical health insurance (34.8%), followed by the Southern region (30.5%), whereas in the North, only 12.7% of the population had private health insurance in 2019. We also found that the Southeast had 54.1% of specialists in head and neck Surgery, having the largest number of professionals trained and qualified to perform these procedures, while the North had only 4% of the total number of specialists or in-country experts. In addition to the discrepant absolute number of professionals, according to the habitational arrangement in a relative survey, the disparity continues, as there are 0.8 head and neck surgeons for every 100,000 inhabitants in the Southeast region and 0.3 specialists per 100,000 citizens in the North (16).

In addition, about the years of thyroidectomies, we observed that the years 2012 and 2020 had the highest and lowest number of surgeries performed, respectively. We also observed that non-oncological thyroidectomies showed a slight drop pattern, while oncological thyroidectomies had a slight increase and the smallest variation in the last year. This is related to the incidence of thyroid cancer, which has grown in recent decades, whether due to exposure to risk factors or increased diagnosis due to the greater use of imaging tests and technological advances, thus also increasing thyroid removal for oncological causes (5,17).

The decrease that occurred in 2020 is related to the SARS-CoV-2 pandemic, which interrupted most of the elective procedures performed by SUS, such as surgeries and outpatient care (18). At that time, there was a need to transfer financial, structural, and professional resources to fight the virus and prevent contamination, especially for patients with comorbidities (18). However, even with the pandemic, the number of oncological thyroidectomies did not have a sharp decline, as this operation presents itself as an essential measure for treatment, and it should be performed as early as possible to prevent the advancement of a common and growing cancer. Early surgery also improves patient survival (19).

Mortality from the procedure is a prominent prism. In the 11 years studied, there were 256 deaths, constituting a mortality rate equal to 16 deaths per 10,000 procedures. The rate had small variations in this period, with a slight tendency to decrease over the years, and in 2019, there was a significant drop in mortality. The highest mortality rate occurred in the Northern region, which is related to the unequal distribution of specialized professionals, resources offered, and advanced centers (15). In addition, the procedure that showed the highest mortality rate was total thyroidectomy with lymph node dissection because it is a more complicated and extensive operation and is linked to cases of more advanced cancers (20).

Another important point is that the value of the procedures is directly proportional to the type of surgery and its quantity. Oncological procedures are up to six times the value of non-oncological procedures, as they are more delicate operations with greater morbidity, and are generally performed in patients with impaired general health. Thus, they are more expensive and demand greater public investments in health, which can make it difficult to carry them out in regions with less health financing, such as the North. Thus, this fact generates an overall average cost of all procedures in the North that is lower than in the rest of the country, since the focus is on surgeries with lower cost, as they can sometimes be performed, even if they are not ideal for the patient (3,15).

As a limitation of the study, underreporting stands out. This is characterized as a weakness in the complete and adequate completion of the SUS information system. In addition, there may be misclassifications such as causes of death not related to the procedure, as well as correct categorization according to codes, with or without association with cancer, apart from the duplication of codes. However, it was possible to conduct the study, which shows the designs of centers whose professionals need more training for notification (21).

In this research, we aim to demonstrate the types of surgeries for thyroid removal specified by the SUS, highlighting the differences between macro-regions, their mortality, the impact of the pandemic, and material and human costs. Moreover, despite the limitations, the primary purpose of this study is to contribute to the literature as a basic subsidy for research addressing surgical treatment and thyroid disorders.

In conclusion, in this study, it was possible to observe that thyroidectomies are performed mainly in the Southeastern, Northeastern, and Southern regions, showing a downward trend in 2020, which may be related to the COVID-19 pandemic. In addition, we confirmed total thyroidectomy as the most commonly performed surgery; the North had the highest mortality rate, and we found that oncological procedures had a higher cost.
